# Balancing effort and benefit of *K*-means clustering algorithms in Big Data realms

**DOI:** 10.1371/journal.pone.0201874

**Published:** 2018-09-05

**Authors:** Joaquín Pérez-Ortega, Nelva Nely Almanza-Ortega, David Romero

**Affiliations:** 1 Departamento de Ciencias Computacionales/Centro Nacional de Investigación y Desarrollo Tecnológico, Tecnológico Nacional de México, Cuernavaca, Morelos, Mexico; 2 Instituto de Matemáticas, Universidad Nacional Autónoma de México, Cuernavaca, Morelos, Mexico; Southwest University, CHINA

## Abstract

In this paper we propose a criterion to balance the processing time and the solution quality of *k*-means cluster algorithms when applied to instances where the number *n* of objects is big. The majority of the known strategies aimed to improve the performance of *k*-means algorithms are related to the initialization or classification steps. In contrast, our criterion applies in the convergence step, namely, the process stops whenever the number of objects that change their assigned cluster at any iteration is lower than a given threshold. Through computer experimentation with synthetic and real instances, we found that a threshold close to 0.03*n* involves a decrease in computing time of about a factor 4/100, yielding solutions whose quality reduces by less than two percent. These findings naturally suggest the usefulness of our criterion in Big Data realms.

## Introduction

The skyrocketing technological advances of our time are enabling a substantial increase in the amount of generated and stored data [1–4], both in public and private institutions, in science, engineering, medicine, finance, education, and transportation, among others. Hence, there is a well justified interest in the quest of useful knowledge that can be extracted from large masses of data, allowing better decision making or improving our understanding of the universe and life.

However, the handling or interrogation of large and complex masses of data with standard tools —termed Big Data— is generally limited by the available computer resourses [1, 5]. In this regard, our contribution here is to provide a strategy to deal with the problem of clustering objects according to their attributes (characteristics, properties, etc.) in Big Data realms.

The clustering problem has long been studied. Its usefulness is indeniable in many areas of human activity, both in science, business, machine learning [6], data mining and knowledge discovery [7, 8], pattern recognition [9], to name but a few. The clustering consists in partitioning a set of *n* objects in *k* ≥ 2 non-empty subsets (called clusters) in such a way that the objects in any cluster have similar attributes and, at the same time, are different from the objects in any other cluster.

In this paper we assume that the objects’ attributes are measurable. Hence, sometimes we refer to objects as points, according to the context.

Let *N* = {*x*_1_, …, *x*_*n*_} denote the set of *n* points to be grouped by a closeness criterion, where *x*_*i*_ ∈ ℜ^*d*^ for *i* = 1, …, *n*, and *d* ≥ 1 is the number of dimensions (the objects’ attributes). Further, let *k* ≥ 2 be an integer and *K* = {1, …, *k*}. For a *k*-partition P={G(1),…,G(k)} of *N*, denote *μ*_*j*_ the centroid of group (cluster) *G*(*j*), for *j* ∈ *K*, and let *M* = {*μ*_1_, …, *μ*_*k*_}.

Thus, the clustering problem can be posed as a constrained optimization one: (see, for instance, [10])

$$\begin{array}{c} P\text{:}\;\text{minimize}\;z\left(W,M\right)=\sum_{i=1}^{n}\sum_{j=1}^{k}w_{ij}d\left(x_{i},\mu_{j}\right)\\ \text{subject}\;\text{to}\;\;\;\sum_{j=1}^{k}w_{ij}=1,\;\text{for}\;i=1,\ldots ,n,\\ w_{ij}=0\;\text{or}\;\text{1,}\;\;\text{for}\;i=1,\ldots ,n\text{,}\;\text{and}\;j=1,\ldots ,k\text{,} \end{array}$$ (1)

where *w*_*ij*_ = 1 ⇔ point *x*_*i*_ belongs to cluster *G*(*j*), and *d*(*x*_*i*_, *μ*_*j*_) denotes the Euclidean distance between *x*_*i*_ and *μ*_*j*_, for *i* = 1, …, *n*, and *j* = 1, …, *k*.

Since the pioneering studies by Steinhaus [11], Lloyd [12], and Jancey [13], many investigations have been devoted to finding a *k*-partition of *N* that solves ***P*** above. It has been proved that this problem belongs to the so-called *NP*-hard class for *k* ≥ 2 or *d* ≥ 2 [14, 15]; thus obtaining an optimal solution of a moderate size instance is in general intractable.

Therefore, a variety of heuristic algorithms have been proposed to approach the optimal solution of ***P***, the most conspicuous being those generally designed as *k*-means [16], with straightforward implementation. It must be said that the establishment of useful gaps between the optimal solution of the problem ***P*** and the solution obtained by *k*-means remains an open problem. The computational complexity of *k*-means is *O*(*nkdr*), where *r* stands for the number of iterations [5, 17], limiting their use in large instances since, in general, at every iteration all distances from objects to cluster centroids must be considered. Thus, numerous strategies to stop iterating have been investigated where, usually, increasing the computational effort entails the reduction of the objective function.

Our aim here is to propose a sound criterion to balance the processing time and the solution quality of *k*-means cluster algorithms in Big Data realms. So, we consider throughout the algorithm K-means—intentionally denoted with capitals to distinguish it from the *k*-means family to which it belongs—, greatly inspired by the seminal ones by Jensey [13] and Loyd [12], the latter appearing as one of the most widely used because of its simplicity and elegance [18]. Obviously, our proposal could be as well applied to most procedures of the *k*-means family.

The rest of the paper proceeds as follows. Section Related work briefly reviews the most relevant investigations known to the authors to improve the efficiency of *k*-means algorithms. Then, the algorithm K-means is described and its behavior is analysed in Section Behavior of K-means, so as to highlight an observed strong correlation between the decreasing value of the objective function (1), and the number of objects changing cluster at every iteration. Consequently, from this situation was born our idea to stop the algorithm in the convergence step, namely, as soon as the number of objects that change cluster is smaller than a given threshold; aimed to balance the processing time and the solution quality, Section Determining threshold values, proposes a procedure to determine this threshold. Next, Section Proposal validation, deals with a validation of our proposal: We computationally tested it on several synthetic and real instances. Finally, Section Conclusion, contains our conclusions.

## Related work

To date, few investigations report theoretical analyzes on *k*-means. Our proposed methodology, as the vast majority of the published improvements for *k*-means, is supported by computer experimentation. Among them, the most relevant are briefly reviewed below, grouped according to the four stages of the algorithm, and adding some related to the choice of the appropriate number of clusters. Undeniably, although some improvements appear to dominate others, from the *No free lunch Theorem* [19] the only way one strategy can outperform another is if it is specialized to the structure of the specific problem under consideration.

### Initialization step

For a successful improvement of the solutions quality, and reducing the number of iterations, Arthur & Vassilvitskii [20] proposed a random generation of the initial *k* centroids as follows. The first centroid is randomly chosen with uniform distribution in *N*; then, for *j* = 2, …, *k*, the probability of a point in *N* to be chosen as the *j*-th centroid is proportional to the square of its minimal distance to the set of 1, …, *j* − 1 already selected centroids.

Zhanguo et al. [21] strategically label the initial centroids, so as to guide the subsequent classification; the maximal distance principle is proposed to advantageously distribute the initial centroids in the solution space. In the paper by Salman et al. [22] the initial centroids are those obtained as final by *k*-means when applied on a 0.1*n*-size, random subset of the *n* objects. Time savings of around 66 percent are reported.

On their part, El Agha & Ashour [23] claim that the following initialization strategy yields improved results. For *s* = 1, …, *d*, and *i* = 1, …, *n*, let *x*_*i*_(*s*) be the *s*-th coordinate of point *x*_*i*_, ${\bar{\nu }}_{s}=\max x_{i}\left(s\right)$, and *ν*_*s*_ = min *x*_*i*_(*s*). Also, let $\nu_{s}=\frac{1}{k}\left({\bar{\nu }}_{s}-{\underline{\nu }}_{s}\right)$. The initial centroids are randomly generated in the main diagonal of the rectangular grid defined by coordinates ${\underline{\nu }}_{s},\;{\underline{\nu }}_{s}+\nu ,\;{\underline{\nu }}_{s}+2\nu ,\ldots ,{\bar{\nu }}_{s}$, for *s* = 1, …, *d*.

Finally, Tzortzis & Aristidis [24] employ maxima and minima values to improve the clustering quality at each iteration, particularly useful when clusters are sought with similar number of objects.

### Classification step

In regard to the classification step, Fahim [25] poses that if the distance of an object, say *x*_*i*_, to the centroid of its assigned cluster, say *G*(*j*), decreases at any iteration, then *x*_*i*_ stays in *G*(*j*), not needing to compute the distance from *x*_*i*_ to the remaining centroids.

Among the various works proposing to apply the principle of the triangle inequality to reduce the number of times that the distance from objects to centroids is computed, the most conspicuous are those by Elkan [18] and Hamerly [26]. By suggesting lower and upper bounds for distances, the former proposes that in every iteration, the distance from any object to any centroid should not be re-calculated if, in the previous iteration, that distance is outside these bounds; it is claimed that with this strategy the efficiency of *k*-means is higher as *k* and *n* increase. In [26] a variant of the Elkan [18] approach is proposed by establishing new bounds on the distances, asserting to improve Elkan results for instances of low dimension.

Pérez and collaborators [27, 28] discuss heuristics to simplify the computation of the distance between objects and centroids. In [28], noting that when objects migrate they normally do so towards a nearby cluster, only the distances from *x* ∈ *N* to the centroids of the *w* clusters closest to *x* are recalculated. Convenient values were experimentally found for *w*, depending on the dimension *d*; so, for instance, *w* = 4, 6 for *d* = 2, 4, respectively.

Two heuristics are considered in [27]; the first one permanently assigns object *x* ∈ *N* to cluster *G*(*j*) as soon as the distance from *x* to *μ*_*j*_ is lower than a given threshold, excluding *x* from subsequent distance computations. In the second heuristic a cluster is labelled ‘stable’ if it has no object exchanges in two successive iterations; objects in stable clusters no longer migrate. A similar heuristic is studied by Lai [29].

Kanungo et al. [30] have theoretically shown that the efficiency of *k*-means is enhanced when a *kd-tree* data structure is used. For Chiang et al. [31] objects very close to their nearest centroid are considered as with negligible probability to change cluster, hence they are excluded from subsequent distance computations.

### Convergence step

The most common convergence criterion of *k*-means is to stop as soon as no change in the clustering is observed. However, it seems advisable to set un upper bound on the number of iterations as a concomitant convergence criterion, as offered by software packages such as SPSS, WEKA, and R.

Denote *z*_*r*_ the objective function value at iteration *r*. Pérez et al. [32] propose to stop the algorithm whenever *z*_*r*_ > *z*_*r*−1_, while in [33] the procedure stops if $|\left(z_{r-1}^{2}-z_{r}^{2}\right)/z_{r}^{2}|<0.001$ along ten successive iterations.

Mexicano et al. [34] compute the largest centroid displacement found in the second iteration (denote it *ψ*). Then, they assume that the *k*-means algorithm has converged if in two successive iterations the largest change of centroids position is lower than 0.05*ψ*.

### Selecting the number *k* of clusters

In the *k*-means realm, the choice of an appropriate *k* value depends on the instances considered, and is a rather difficult task; this situation is usually addressed by trial and error. However, several investigations have been carried out to automatically determine the number *k* of clusters; see, for example, those reported in [35, 36], and the more recent approaches [37–39] that employ a Bayesian nonparametric view.

## Behavior of K-means

We made intensive computational experiments to assess the behavior of the below algorithm K-means under different conditions.

**Algorithm** K-means

**Step 1 Initialization**. Produce points *μ*_1_,…,*μ*_*k*_, as a random subset of *N*.

**Step 2 Classification**. For all *x* ∈ *N* and *j* ∈ *K*, compute the Euclidean distance between points *x* and *μ*_*j*_, namely, *d*(*x*, *μ*_*j*_). Then, point (object) *x* ∈ *N* is assigned to a cluster $G(\ddot{ȷ})$ if $d\left(x,\mu_{\ddot{ȷ}}\right)\leq d\left(x,\mu_{j}\right)$, for $\ddot{j},j\in K$.

**Step 3 Centroids**. Determine the centroid *μ*_*j*_ of cluster *G*(*j*), for *j* ∈ *K*.

**Step 4 Convergence**. If the set of centroids *M* does not change in two successive iterations stop the algorithm, otherwise perform another iteration starting from Step 2.

In general, a correlation was observed between the number of objects changing cluster at each iteration, and the corresponding *z* value of the objective function (1).

As an example of what we found, Table 1 displays some results yielded by a run of K-means on a synthetic instance with *n* = 2 ⋅ 10^6^ points (objects) randomly generated in the unit square (uniform distribution), and *k* = 200.

**Table 1:** Some results of the K-means algorithm with a randomly generated instance where *n* = 2 ⋅ 10^6^, *k* = 200, and *d* = 2.

*r*	*z_r_*	*γ_r_*	*δ_r_*
**1**	63 187	–	17.62
**2**	58 819	15.88	9.48
**3**	57 238	9.22	6.54
**4**	56 458	6.32	5.09
**5**	55 985	4.77	4.21
**6**	55 656	3.88	3.60
**7**	55 417	3.28	3.15
**8**	55 235	2.87	2.81
**9**	55 090	2.56	2.54
**10**	54 971	2.32	2.32
**11**	54 871	2.09	2.14
**12**	54 787	1.89	1.98
**13**	54 715	1.75	1.84
**14**	54 652	1.61	1.73
**15**	54 598	1.48	1.63
**16**	54 550	1.38	1.54
**17**	54 507	1.31	1.46
**18**	54 468	1.23	1.39
**19**	54 433	1.17	1.32
**20**	54 401	1.10	1.26
**21**	54 373	1.04	1.21
**22**	54 348	0.98	1.16
**23**	54 327	0.92	1.12
**24**	54 306	0.88	1.08
**25**	54 288	0.83	1.05
**26**	54 271	0.80	1.02
**27**	54 256	0.77	0.99
**28**	54 241	0.75	0.96
**29**	54 226	0.74	0.93
**30**	**54 211**	**0.72**	**0.91**
**31**	54 197	0.70	0.88
**32**	54 183	0.69	0.85
**33**	54 169	0.66	0.83
**34**	54 157	0.64	0.81
**35**	54 146	0.61	0.79
…	…	…	…
…	…	…	…
…	…	…	…
**612**	53 721	0	0

The algorithm stops at iteration 612 because no points change cluster.

Each row of Table 1 contains information corresponding to iteration *r*: The objective function value *z*_*r*_ (we denote z* the lowest value found by the algorithm, thus, z* = *z*_612_ = 53721), the indicator *γ*_*r*_ = 100(*υ*_*r*_/*n*), where *υ*_*r*_ is the number of points changing cluster, and *δ*_*r*_ = 100(*z*_*r*_/z* − 1).

**Fig 1:**
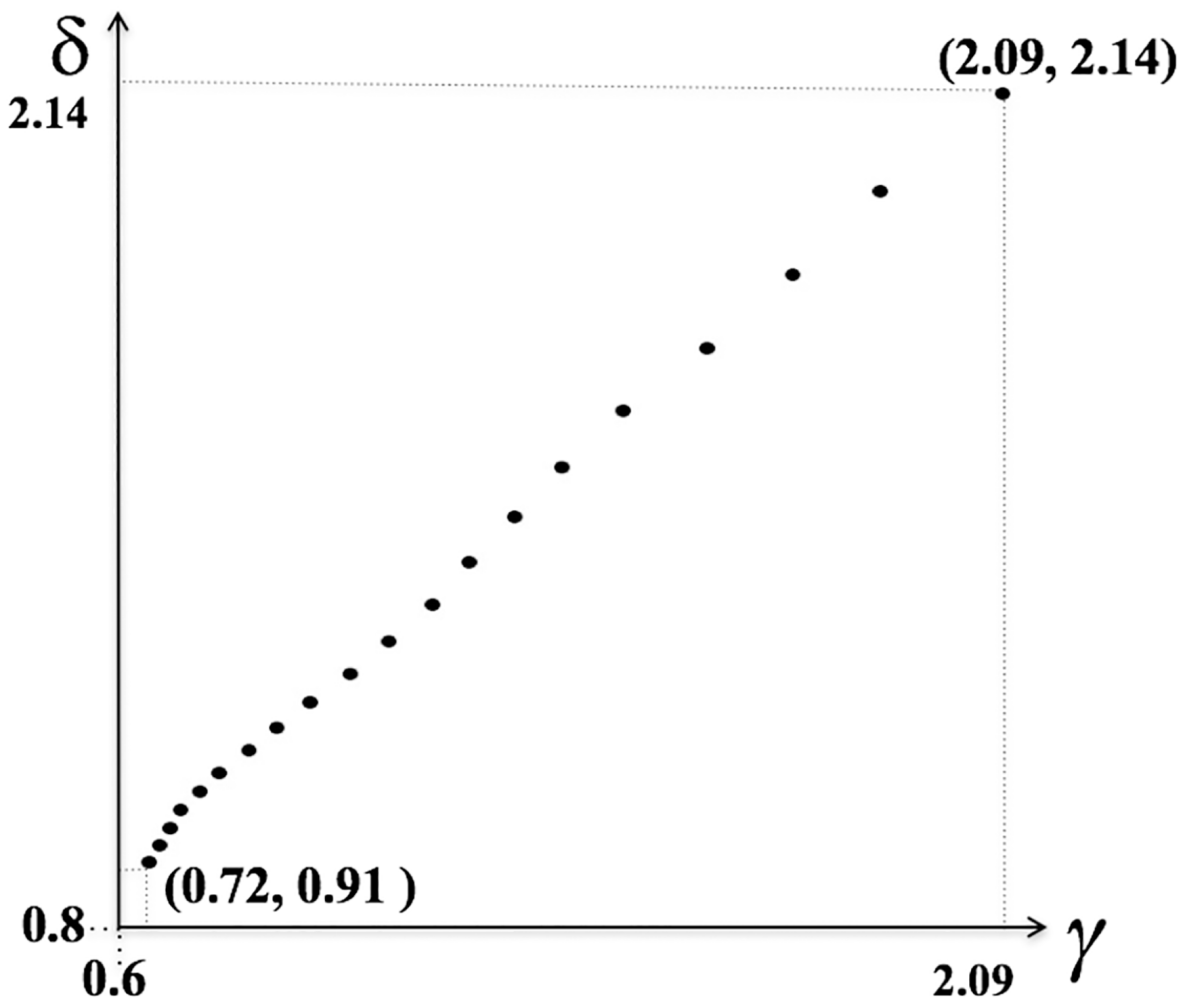
An almost linear relationship between *γ* and *δ*.

**Fig 2:**
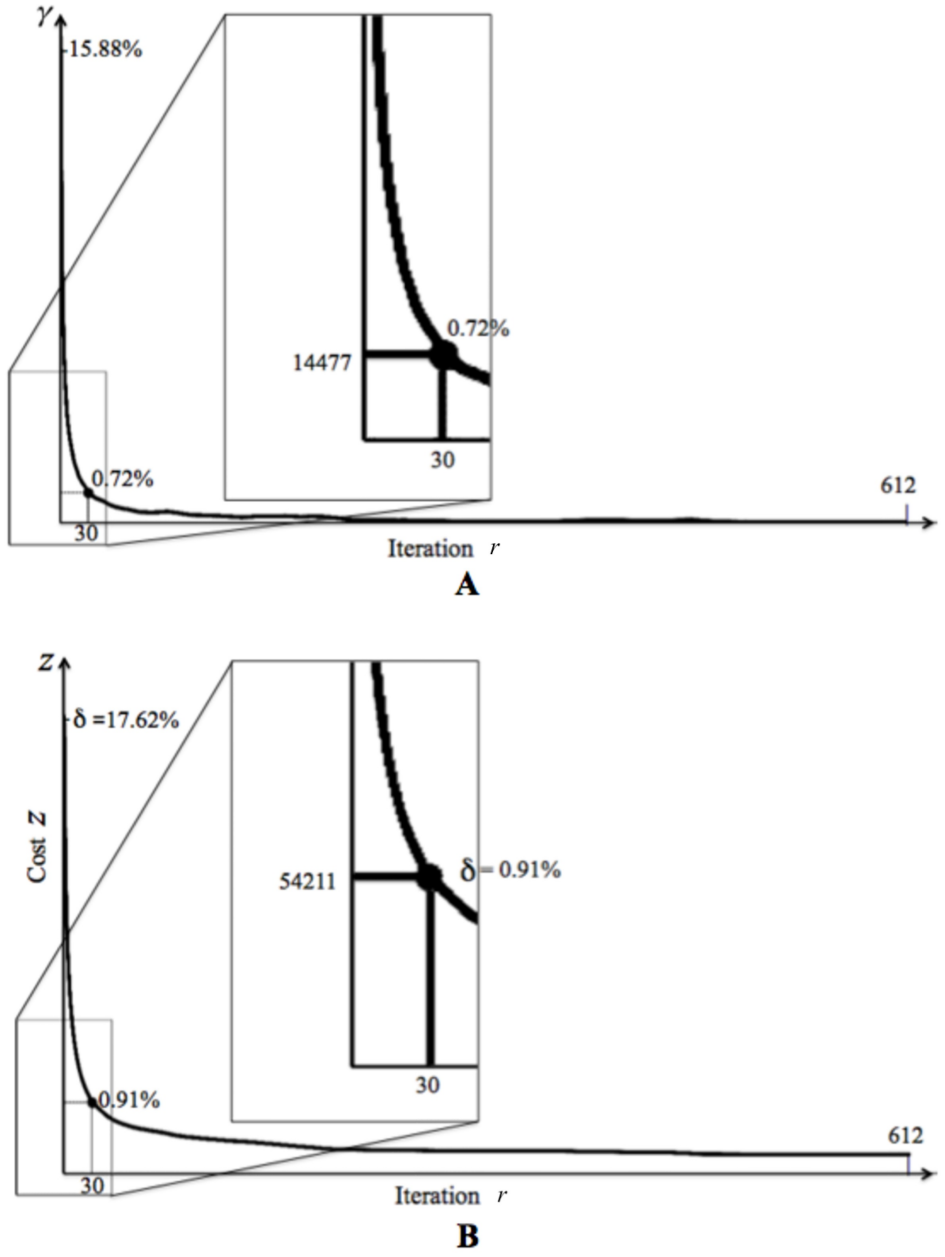
Graphics of *γ* and *δ* per iteration. (A) Percentage of objects changing cluster per iteration. (B) Cost *z* per iteration.

To grasp the algorithm behavior from Table 1 consider Fig 1—for *r* = 11, …, 30, points (*γ*_*r*_, *δ*_*r*_) lie in an almost straight line— as well as the striking similarity of curves in Fig 2.

This clearly suggests a strong correlation between indexes *γ* and *δ*, and between *z* and *γ*. Hence, letting *ℓ* be the total number of iterations, $\bar{\gamma }=\frac{1}{\ell }\sum_{r=2}^{\ell }\gamma_{r}$, and $\bar{\delta }=\frac{1}{\ell }\sum_{r=2}^{\ell }\delta_{r}$, the correlation coefficient between *γ* and *δ* is calculated with the Eq (2) thus verifying our assumptions.

$$\begin{array}{c} \rho \left(\gamma ,\delta \right)=\frac{\sum_{r=2}^{\ell }\left[\left(\gamma_{r}-\bar{\gamma }\right)\left(\delta_{r}-\bar{\delta }\right)\right]}{\sqrt{\sum_{r=2}^{\ell }{\left(\gamma_{r}-\bar{\gamma }\right)}^{2}}\sqrt{\sum_{r=2}^{\ell }{\left(\delta_{r}-\bar{\delta }\right)}^{2}}}=0.96 \end{array}$$ (2)

A similar experiment was made with a set of real and synthetic instances —described in Section Proposal validation— obtaining the correlation coefficients as shown in Table 2. Note that no value is below 0.9, and most lie in the range [0.975, 0.998].

**Table 2:** Correlation coefficients of synthetic and real instances.

Synthetic instance	d	k	Correlation coefficient
**1**	2	200	0.966
**2**	4	100	0.989
**3**	6	100	0.996
**4**	5	200	0.976
**6**	2	50	0.990
**6**	2	100	0.984
**6**	2	200	0.984
**7**	3	50	0.989
**7**	3	100	0.989
**7**	3	200	0.976
**8**	4	50	0.992
**8**	4	100	0.988
**8**	4	200	0.975
**9**	7	200	0.994
**10**	10	100	0.966
**11**	2	50	0.980
**12**	11	100	0.930
**13**	12	50	0.914
**14**	5	100	0.998
**14**	5	200	0.991
Real instance	d	k	Correlation
**1**	7	20	0.980
**1**	7	50	0.974
**2**	15	160	0.979
**2**	15	80	0.992
**3**	16	50	0.991
**4**	45	100	0.987
**4**	45	50	0.992
**5**	7	100	0.950
**5**	7	50	0.904

The aforementioned considerations led us to our proposal of a criterion to balance the computational effort and the solution quality for *k*-means in Big Data realms. Section Determining threshold values, describes the path we followed to determine threshold values to be used in the convergence step so as to judiciously stop the algorithm.

## Determining threshold values

Although the computational effort at each iteration of *k*-means is rather constant, the corresponding improvement on *z* is not. Thus the question: When is it worth to keep iterating? It is well known that the so-called *Pareto principle* can help in determining an optimal relationship between effort and benefit [40]. Thus, we relied on this principle to provide a sound answer to the above question.

Continuing with the example of Section Behavior of K-means, we computed Table 3 where, for *r* ≥ 2, *A*_*r*_ = 100(*r*/*ℓ*), *B*_*r*_ = 100(*z*_*r*−1_ − *z*_*r*_)/(*z*_1_ − z*), *C*_*r*_ = *C*_*r*−1_ + *B*_*r*_, and $D_{r}=\sqrt{{\left(0-A_{r}\right)}^{2}+{\left(100-C_{r}\right)}^{2}}$ is the Euclidean distance between points (*A*_*r*_, *C*_*r*_) and (0, 100).

**Table 3:** Information to seize the relationship between computational effort and solution quality.

*r*	*A_r_*	*B_r_*	*C_r_*	*D_r_*
**1**	0.163			
**2**	0.326	46.150	46.150	53.850
**3**	0.490	16.701	62.852	37.151
**4**	0.653	8.235	71.087	28.919
**5**	0.816	4.995	76.082	23.931
**6**	0.980	3.471	79.554	20.468
**7**	1.143	2.533	82.088	17.948
**8**	1.307	1.922	84.011	16.042
**9**	1.470	1.525	85.536	14.538
**10**	1.633	1.263	86.799	13.301
**11**	1.797	1.053	87.853	12.278
**12**	1.960	0.886	88.739	11.430
**13**	2.124	0.765	89.504	10.707
**14**	2.287	0.660	90.165	10.097
**15**	2.450	0.568	90.733	9.584
**16**	2.614	0.505	91.238	9.142
**17**	2.777	0.455	91.694	8.757
**18**	2.941	0.411	92.106	8.423
**19**	3.104	0.377	92.483	8.132
**20**	3.267	0.334	92.817	7.890
**21**	3.431	0.296	93.114	7.693
**22**	3.594	0.258	93.372	7.539
**23**	3.758	0.230	93.603	7.419
**24**	3.921	0.213	93.816	7.322
**25**	4.084	0.193	94.009	7.250
**26**	4.248	0.178	94.188	7.198
**27**	4.411	0.163	94.351	7.166
**28**	4.575	0.159	94.511	7.145
**29**	4.738	0.156	94.668	7.133
**30**	**4.901**	**0.152**	**94.820**	**7.131**
**31**	5.065	0.152	94.973	7.136
**32**	5.228	0.150	95.123	7.149
**33**	5.392	0.139	95.263	7.177
**34**	5.555	0.132	95.395	7.215
**35**	5.718	0.119	95.515	7.267
…	…	…	…	…
…	…	…	…	…
**612**	100	0	100	100

**Fig 3:**
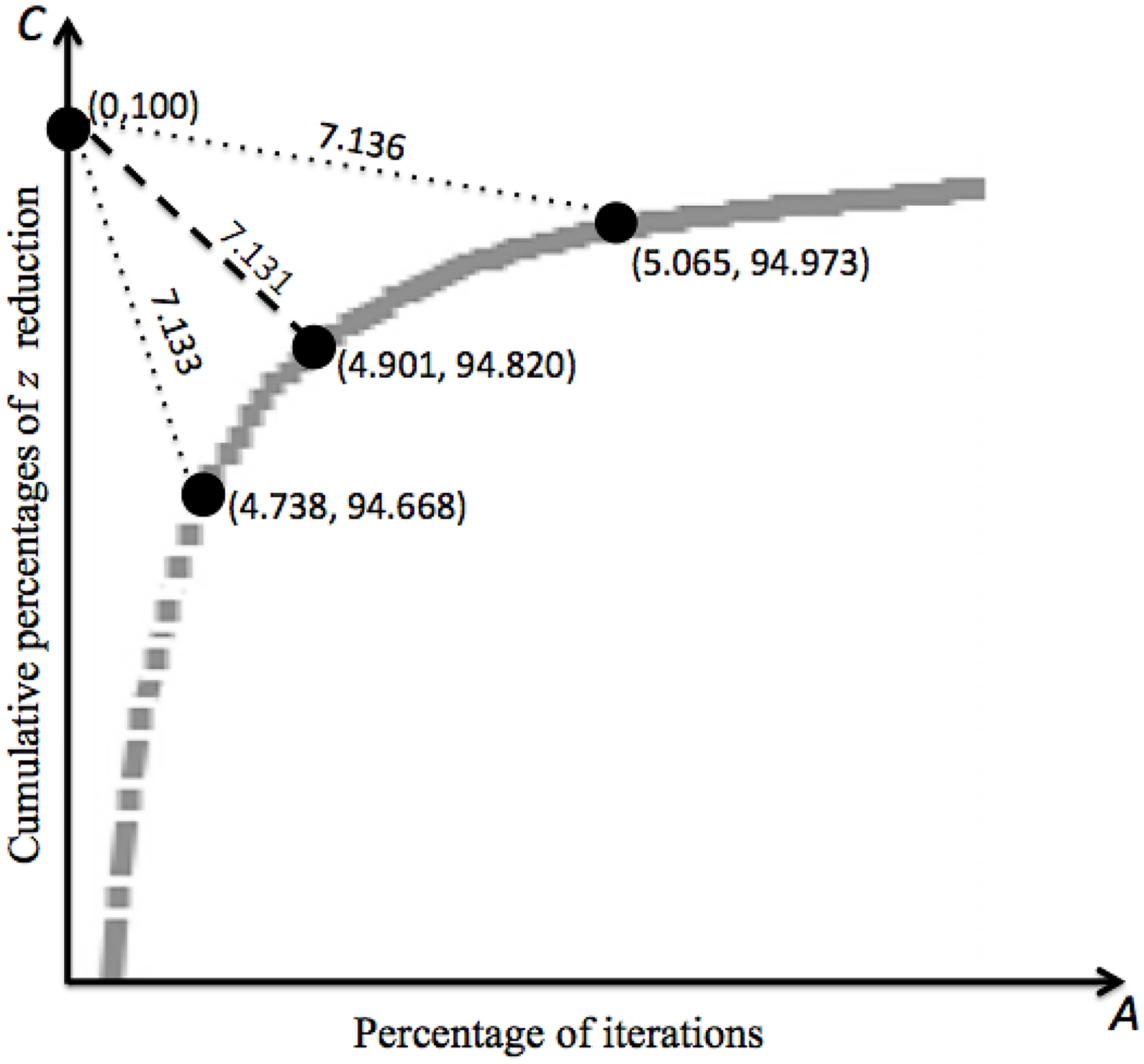
Pareto optimality.

Fig 3 shows a partial plot of the Pareto diagram for points (*A*_*r*_, *C*_*r*_) extracted from Table 3. Note that *D*_30_ ≤ *D*_*r*_ for any *r* ∈ *N*, namely, (*A*_30_, *C*_30_) is the closest point to (0,100). Hence, we could stop K-means at iteration *r* = 30 = 0.0490*ℓ*, avoiding as much as ≈95% of iterations, to get a solution with cost *z*_30_ = 1.0091z* (*δ*_30_ = 0.91 comes from Table 1), namely, only less than one percent worst than z*. Also, observe in Table 1 that at iteration 30 as few as 0.0072*n* objects migrate.

Following the ideas and concepts discussed over the previous example, we undertook intensive computer experimentation applying the Pareto principle on a variety of instances. As a result we obtained a set of threshold values *U* such that, depending on the available computer time, on the instance size, and according to the needs, it seems reasonable to stop K-means at iteration *r* whenever *γ*_*r*_ ≤ *U*. Recall *γ*_*r*_ = 100(*υ*_*r*_/*n*), where *υ*_*r*_ is the number of migrating points at iteration *r*.

The algorithm below, which we call *O*-K-means (optimized K-means), results when our criterion is incorporated into K-means.

*O*-K-means

**Step 1 Initialization**. Produce points *μ*_1_,…,*μ*_*k*_, as a random subset of *N*.

**Step 2 Classification**. For all *x* ∈ *N* and *j* ∈ *K*, compute the Euclidean distance between points *x* and *μ*_*j*_, namely, *d*(*x*, *μ*_*j*_). Then, point (object) *x* ∈ *N* is assigned to a cluster $G(\ddot{ȷ})$ if $d\left(x,\mu_{\ddot{ȷ}}\right)\leq d\left(x,\mu_{j}\right)$, for $\ddot{ȷ},j\in K$.

**Step 3 Centroids**. Determine the centroid *μ*_*j*_ of cluster *G*(*j*), for *j* ∈ *K*.

**Step 4 Convergence**. If *γ*_*r*_ ≤ *U* stop the algorithm, otherwise perform another iteration starting from Step 2.

## Proposal validation

By means of C language and a GCC 4.9.2 compiler, both K-means and *O*-K-means algorithms were implemented on a Mac mini computer with OS Yosemite 10.10, processor Core i5 at 2.8GHz, and 16 GB of RAM memory. The implementation of our stopping strategy requires negligible additional memory resources.

For the design of the computational experiments and the analysis of our algorithms, we used the methodology proposed by McGeoch [41].

As mentioned in Section Introduction the complexity of K-means is *O*(*nkdr*). Therefore, the *O*-K-means can be expressed as *O*(*nkdrα*), where *α* denotes the quotient of the number of *O*-K-means iterations and the number of K-means iterations. From our experiments with large synthetic and real instances we obtained an average of *α* = 0.0389.

Our codes were tested on sets of real and synthetic instances. In each instance the initial centroids were the same for both algorithms. In what follows we denote *t*, *t*_*o*_, z*, and $z_{o}^{*}$, respectively, the time needed by K-means, the time needed by *O*-K-means, the best objective function value found by K-means, and the best objective function value obtained by *O*-K-means. Further, to mesure time saving and quality loss we use, respectively, the formulas

$$\begin{array}{c} \delta =100(1-\frac{z^{*}}{z_{o}^{*}})\;\;\text{and}\;\;\rho =100(1-\frac{t_{o}}{t}). \end{array}$$

For each instance we chose the product *nkd* as an indicator of its complexity level. The computer experiments described in sections Synthetic instances and Real instances used different *U* values according to the Pareto optimality principle applied to each instance. Consideration of different thresholds for the same instance is dealt with in Section Using other threshold values.

### Synthetic instances

14 synthetic instances were produced for different *n* values and number of dimensions *d* as shown in Table 4.

**Table 4:** Synthetic instances general data. The *n* values are given in millions.

Instance	1	2	3	4	5	6	7	8	9	10	11	12	13	14
*n*	2	2	2	2	2	1	1	1	1	1	0.5	0.5	0.25	1.2
*d*	2	4	6	5	7	2	3	4	7	10	2	11	12	5

All points in each synthetic instance were randomly generated: while in instances 1 to 13 they followed a uniform distribution with coordinates belonging to (0, 1)^*d*^, in instance 14 a normal distribution was used with mean 40 and standard deviation 20. Distinct values for the number of clusters *k* were considered. Table 5 shows the results of an execution of each algorithm; the computing times *t* and *t*_*o*_ are reported in hours rounded to hundredths. Notice that the instances are sorted according to the *nkd* product.

**Table 5:** Results of K-means and *O*-K-means on synthetic instances. Computer times *t* and *t*_*o*_ are given in hours, the product *nkd* in millions.

Instance	*k*	*nkd*	*t*	*t*_*o*_	z*	zo*|||	*ρ* (%)	*δ* (%)	*U*
**11**	50	50	0.38	0.01	26 959	27 140	96.19	0.671	1.23
**6**	50	100	1.28	0.02	53 988	54 148	97.82	0.296	0.84
**7**	50	150	0.53	0.03	130 439	131 249	93.31	0.620	1.36
**13**	50	150	1.14	0.06	188 184	188 705	94.62	0.276	1.01
**6**	100	200	1.52	0.07	38 065	38 336	95.38	0.712	0.96
**8**	50	200	2.00	0.07	211 690	213 294	96.42	0.757	0.73
**7**	100	300	2.62	0.09	103 010	103 611	96.40	0.583	0.77
**6**	200	400	2.91	0.13	26 848	27 084	95.42	0.879	0.87
**8**	100	400	1.54	0.08	177 224	178 534	94.19	0.739	1.47
**12**	100	550	9.08	0.37	324 023	324 825	95.89	0.247	0.63
**7**	200	600	6.17	0.22	81 418	81 823	96.28	0.497	0.78
**14**	100	600	7.41	0.18	28 606 830	28 719 403	97.49	0.393	1.14
**1**	200	800	8.09	0.39	53 722	54 212	95.10	0.912	0.72
**8**	200	800	9.68	0.37	147 867	148 588	96.08	0.487	0.63
**10**	100	1000	18.56	0.64	588 590	589 796	96.55	0.204	0.73
**14**	200	1200	24.03	0.52	25 001 825	25 096 243	97.81	0.377	0.89
**9**	200	1400	20.30	0.57	353 458	354 444	97.19	0.278	0.79
**2**	100	1600	6.15	0.27	354 738	356 783	95.47	0.576	0.89
**4**	200	2000	26.14	0.79	434 530	435 764	96.94	0.283	0.82
**3**	100	2400	11.49	0.38	648 108	650 778	96.65	0.412	0.87
**5**	100	2800	13.66	0.41	776 098	779 474	96.98	0.434	0.93
**Average**							**96.10**	**0.507**	**0.91**
**Standard deviation**							**1.17**	**0.214**	**0.22**

When compared to K-means, algorithm *O*-K-means exhibits good performance on these instances: on average, in less than four percent of calculation time solutions are obtained whose quality decreases only about 0.5%. It is worth mentioning the relatively low standard deviation value (1.17), as compared to the average (96.10).

### Real instances

We made computer experimentation with five real instances taken from the University of California repository, UCI [42], see Table 6. Note the relatively small size of instances 1–3 when compared to the large instances 4 and 5.

**Table 6:** Real instances general data.

Instance	Name	*n*	*d*
**1**	Abalone	4 177	7
**2**	Wind	6 574	15
**3**	Letters	20 000	16
**4**	DSAS	1 140 000	45
**5**	EPCS	2 075 259	7

For distinct *k* values, each real instance was solved with K-means and *O*-K-means using the same initial centroids; results are shown in Table 7. In general, as *nkd* increases, the efficiency of *O*-K-means increases —note the average time reduction for the large instances (96.12%) and the small ones (86.47%), yielding similar quality loss—. As in the case of synthetic instances, low standard deviations are observed.

**Table 7:** Comparative results for real instances. The product *nkd* is given in millions, and z*, $z_{o}^{*}$ have been rounded to integers. Computing times *t*, *t*_*o*_ are in seconds for the small instances, and in hours for the large ones.

Instance	*k*	*nkd*	*t*	*t*_*o*_	z*	zo*|||	*ρ* (%)	*δ* (%)	*U*
**1**	20	0.58	0.23	0.04	314	320	83.33	1.990	2.22
**1**	50	1.46	0.43	0.05	243	248	87.18	2.002	2.01
**2**	80	7.88	2.60	0.38	57 633	58 245	85.42	1.062	3.84
**2**	160	15.77	4.75	0.65	52 600	53 134	86.36	1.015	4.12
**3**	50	16.00	5.42	0.63	95 283	96 602	88.46	1.384	2.90
**3**	100	32.00	10.53	1.26	81 856	83 044	88.06	1.451	3.75
**Average**							**86.47**	**1.484**	**3.14**
**Standard deviation**							**1.89**	**0.432**	**0.89**
**5**	50	399.00	2.09	0.11	8 878 334	8 935 921	94.84	3.056	3.17
**5**	100	798.00	6.82	0.13	10 018 910	10 073 027	98.04	2.191	4.65
**4**	50	2 565.00	8.17	0.35	2 533 399	2 588 922	95.75	0.540	3.20
**4**	100	5 130.00	16.32	0.68	3 257 026	3 356 560	95.85	0.648	1.76
**Average**							**96.12**	**1.610**	**3.20**
**Standard deviation**							**1.35**	**1.224**	**1.18**

### Using other threshold values

In sections Synthetic instances and Real instances, we showed results for thresholds determined following the Pareto optimality principle; however, other threshold values could as well be selected according to the need of solution quality and time availability.

Thus, each instance of Table 4 was solved with K-means and *O*-K-means using the same initial centroids, and the threshold values *U* shown in Table 8. In this table $\bar{\rho }$ and $\bar{\delta }$ denote the averaged reduction of time and solution quality, respectively. As expected, each row decreases monotonically from left to right, namely, increasing *U* leads to greater reduction of both computing time and quality solution. Thus, there is a trade-off between these concepts.

**Table 8:** Average reduction of computing time $\bar{\rho }$ and solution quality $\bar{\delta }$, obtained by *O*-K-means for distinct threshold values *U*.

*U*	2.5	2	1.5	1	0.5	0.4	0.3	0.2
$\bar{\rho }$	98.31	97.93	97.30	96.23	93.31	91.23	88.71	84.18
$\bar{\delta }$	1.26	0.98	0.74	0.51	0.30	0.23	0.18	0.11

We find remarkable the closeness between the reductions with *U* = 1, and those that arise when applying the Pareto principle (see Table 5).

### Combining our convergence criterion with other criteria

To assess the benefit of combining our convergence criterion with other algorithms for speeding up *k*-means we considered two efficient classification strategies. One was proposed by Fahim et al. [25], call it F, the other is due to Pérez et al. [43], call it P. With this aim we chose 10 synthetic instances described in Table 4, to be solved for distinct *k* values. For each instance the developed codes used the same initial centroids.

Table 9 summarizes our results, in terms of time and quality reduction related to K-means, where OK, FOK, and POK, denote, respectively, *O*-K-means, Fahim et al. algorithm combined with *O*-K-means, and Pérez et al. algorithm combined with *O*-K-means.

**Table 9:** Tests on selected synthetic instances. Time and quality reduction arising from combining F and P algorithms with *O*-K-means.

Instance	*d*	*k*	Time reduction (%)					Quality reduction (%)				
			OK	F	P	FOK	POK	OK	F	P	FOK	POK
**1**	2	200	95.31	75.47	99.06	97.97	99.26	0.67	-0.06	3.06	1.26	3.10
**2**	4	100	93.53	31.18	98.39	96.82	98.80	0.40	-0.03	1.63	0.70	1.65
**4**	5	200	96.66	73.72	98.95	98.18	99.21	0.25	0.03	1.19	0.40	1.21
**5**	7	100	96.35	70.72	98.21	97.96	98.69	0.40	0	0.94	0.47	0.95
**6**	2	50	97.61	85.24	99.12	98.23	99.31	0.24	-0.08	1.95	0.48	1.98
**6**	2	100	94.50	67.22	98.64	97.39	98.89	0.57	0.20	3.17	0.94	3.20
**6**	2	200	94.55	64.71	98.63	97.19	98.90	0.73	-0.08	2.74	1.08	2.77
**7**	3	50	89.21	59.55	96.88	95.18	97.71	0.22	0.06	1.32	0.54	1.34
**7**	3	100	96.10	59.41	98.68	97.93	99.02	0.55	-0.04	1.79	0.83	1.81
**7**	3	200	96.04	73.66	98.95	97.99	99.17	0.46	0.02	1.81	0.70	1.83
**8**	4	50	96.29	57.25	98.75	97.87	99.02	0.74	0	1.74	0.80	1.75
**8**	4	200	96.58	70.38	99.01	98.27	99.24	0.53	0.05	1.49	0.69	1.52
**9**	7	200	96.94	68.43	98.97	98.49	99.19	0.26	0	0.67	0.36	0.68
**10**	10	100	96.48	62.07	99.11	98.45	99.32	0.20	0	0.87	0.33	0.88
**14**	5	200	97.15	75.73	99.27	98.79	99.42	0.30	0.03	0.87	0.44	0.88
**averages**			95.55	66.32	98.71	97.78	99.01	0.43	0.01	1.68	0.67	1.70

The results of our computational experiments lead us to pose that it can be advantageous to combine *O*-K-means with other strategies, both in terms of computing time reduction and solution quality.

**Fig 4:**
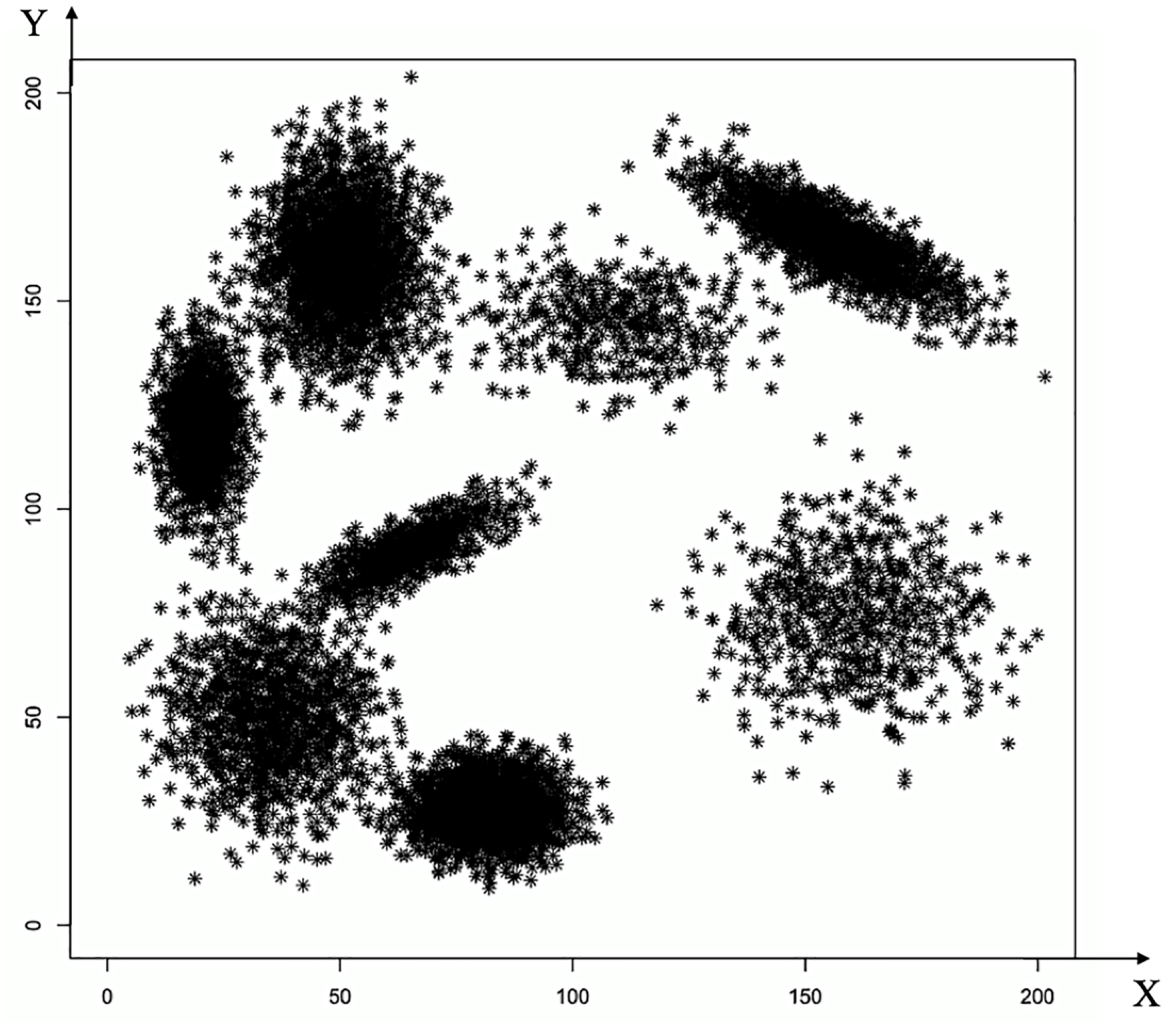
Non uniform synthetic instance in two dimensions.

Also, to assess the algorithms OK, FOK, and POK when using as initial centroids those generated by the algorithm K++ proposed by Arthur [20] we selected eight instances, see Table 10. Instance **A** is the synthetic one shown in Fig 4; instances **B** and **C** correspond to the real instance Letters described in Table 6; instance **D** was produced by randomly selecting 30 000 objects from instance 10 of Table 4; to produce instances **E** and **F** 40 000 points were randomly generated (uniform distribution) in a bounded space; finally, instances **G** and **H** correspond to the instance 10 of Table 4. Table 10 shows the time and quality reduction related to the algorithm K++, obtained by algorithms OK, FOK, and POK when using as initial centroids those generated by K++. These results support our belief in the usefulness of our proposal.

**Table 10:** Results of OK, FOK and POK when using as initial centroids those generated by K++.

Instance	*n*	*d*	*k*	Time reduction (%)			Quality reduction (%)		
				OK	FOK	POK	OK	FOK	POK
**A**	11 000	2	16	74.42	84.92	88.22	0.81	1.05	7.50
**B**	20 000	16	50	91.35	94.50	92.77	1.27	1.89	1.42
**C**	20 000	16	100	81.87	89.50	82.88	1.72	1.42	1.73
**D**	30 000	10	50	81.46	89.64	94.94	0.32	0.42	0.81
**E**	40 000	3	50	77.72	85.84	92.94	0.24	0.53	1.67
**F**	40 000	3	100	79.83	88.08	94.19	0.82	1.09	2.47
**G**	1 000 000	10	50	96.18	97.69	98.99	0.15	0.20	0.53
**H**	1 000 000	10	100	97.75	98.48	99.14	0.28	0.36	0.85

### Data clustered around specific centers

To size up the performance of our stopping criterion if the data is heavily clustered around few specific centers, we constructed a 2-dimensional synthetic instance (*d* = 2), where *n* = 11000 points display compact groups, see Fig 4. Each group was formed by arbitrarily selecting a center and standard deviation, and points were randomly generated with normal distribution around the centers.

This instance was solved for *k* = 5, 10, 15, 20. Table 11 shows the average results of 30 runs of K-means and *O*-K-means with threshold *U* = 3.14, as well as the average reduction of time and quality due by the latter.

**Table 11:** Results of *O*-K-means for a 2-dimensional, non uniform synthetic instance.

*k*	average number of iterations		average reduction	
	K-means	*O*-K-means	time (%)	quality (%)
5	14.23	4.87	64.58	3.90
10	22.20	5.23	75.13	2.90
15	40.10	5.60	85.12	2.86
20	47.43	6.10	86.44	2.86

Note that the best results are obtained as *k* increases, with a time reduction of up to 86.44%, and an average quality reduction as low as 2.86%.

### Clustering larger data sets

We tested the performance of *O*-K-means on four still larger instances, selected from the repository UCI [42]. Table 12 shows their relevant data as well as the average computational results obtained from 30 runs of *O*-K-means, with the same threshold (3.20) used for the real instances of Table 6. These results confirm the suitability of our proposal in Big Data realms.

**Table 12:** Results of *O*-K-means for still larger instances.

Instance	*n*	*d*	*k*	average number of iterations		average reduction	
				K-means	*O*-K-means	time (%)	quality (%)
Arcene	900	10 000	80	10.66	4.03	62.74	0.36
Madelon	1 600	64	100	11.03	3.83	65.46	0.05
Semelon	1 593	256	150	9.23	3.96	60.28	0.21
KDDcup	4 898 432	38	20	104.90	15.53	85.00	5.20

## Conclusion

We have presented a sound criterion to balance effort and benefit of *k*-means cluster algorithms in Big Data realms. Its advantages were demonstrated by applying it in the convergence step of one of the most widely used procedures of the *k*-means family, which here we called K-means. Guided by the Pareto principle, our criterion consists in stopping the iterations as soon as the number of objects that change cluster at any iteration is lower than a prescribed threshold. The novelty of methodology comes from two facts. First, in regard to the stopping criterion, the authors are not aware of any proposal directly related to the number of objects changing group at every iteration. Second, to date the Pareto principle has not been used to determine a threshold leading to an adequate commitment between the quality of a solution and the needed time to obtain it.

From intensive computer experimentation on synthetic and real instances we found that, in general, our criterion significantly reduces the number of iterations with relatively small decrement in the quality of the yielded solutions. Furthermore, the best results tend to correspond to the largest instances considered, namely, those where the product *nkd* is high. Thus, this behavior is an indicator of the usefulness of applying the Pareto principle in the convergence step when dealing with large *k*-means instances.

It is well known that some strategies to improve the performance of *k*-means are sensitive to the number of dimensions. This is not our case, since our proposal aims to reduce the number of iterations made, and the time complexity per iteration *nkd* is taken as a constant.

An important characteristic of our stopping strategy is that its implementation requires negligible additional memory resources; in this regard, it appears to take advantage over other proposed criteria.

Last, but not least, our proposed convergence criterion is not incompatible with any improvement related to the initialization or classification steps of *k*-means, as we have shown in relation to the procedure that generates the initial centroids of the K++ algorithm. As future work we find it appealing to deepen these investigations in the realm of parallel and distributed computing paradigms. It is foreseeable that our stopping criterion can be successfully used under such paradigms since it only requires the number of objects changing group at each iteration.
